# The differential diagnosis of children with joint hypermobility: a review of the literature

**DOI:** 10.1186/1546-0096-7-1

**Published:** 2009-01-05

**Authors:** Louise J Tofts, Elizabeth J Elliott, Craig Munns, Verity Pacey, David O Sillence

**Affiliations:** 1 The Connective Tissue Dysplasia Clinic, The Children's Hospital at Westmead, Sydney, NSW, Australia; 2 Children's Hospital Institute of Sports Medicine, The Children's Hospital at Westmead, Sydney, NSW, Australia; 3 Rehabilitation Department, The Children's Hospital at Westmead, Sydney, NSW, Australia; 4 Discipline of Paediatrics and Child Health, University of Sydney, NSW, Australia; 5 Division of Medicine, The Children's Hospital at Westmead, Sydney, NSW, Australia; 6 Australian Paediatric Surveillance Unit, The Children's Hospital at Westmead, Sydney, NSW, Australia; 7 Department of Endocrinology, The Children's Hospital at Westmead, Sydney, NSW, Australia; 8 Physiotherapy Department, The Children's Hospital at Westmead, Sydney, NSW, Australia; 9 Department of Clinical Genetics, The Children's Hospital at Westmead, Sydney, NSW, Australia

## Abstract

**Background:**

In this study we aimed to identify and review publications relating to the diagnosis of joint hypermobility and instability and develop an evidence based approach to the diagnosis of children presenting with joint hypermobility and related symptoms.

**Methods:**

We searched Medline for papers with an emphasis on the diagnosis of joint hypermobility, including Heritable Disorders of Connective Tissue (HDCT).

**Results:**

3330 papers were identified: 1534 pertained to instability of a particular joint; 1666 related to the diagnosis of Ehlers Danlos syndromes and 330 related to joint hypermobility.

There are inconsistencies in the literature on joint hypermobility and how it relates to and overlaps with milder forms of HDCT. There is no reliable method of differentiating between Joint Hypermobility Syndrome, familial articular hypermobility and Ehlers-Danlos syndrome (hypermobile type), suggesting these three disorders may be different manifestations of the same spectrum of disorders. We describe our approach to children presenting with joint hypermobility and the published evidence and expert opinion on which this is based.

**Conclusion:**

There is value in identifying both the underlying genetic cause of joint hypermobility in an individual child and those hypermobile children who have symptoms such as pain and fatigue and might benefit from multidisciplinary rehabilitation management.

Every effort should be made to diagnose the underlying disorder responsible for joint hypermobility which may only become apparent over time. We recommend that the term "Joint Hypermobility Syndrome" is used for children with symptomatic joint hypermobility resulting from any underlying HDCT and that these children are best described using **both **the term Joint Hypermobility Syndrome **and **their HDCT diagnosis.

## Background

In this review we aimed to identify the current literature pertaining to the diagnosis of children with joint hypermobility. The focus is on clinical signs or investigations which reliably allow children with a Heritable Disorder of Connective Tissue (HDCT) and mild musculoskeletal impairment to be distinguished from children who fall within the normal spectrum of joint mobility.

Joint hypermobility is common in childhood, occurring in 8–39% of school age children[1-4]. Prevalence depends on age, sex and ethnicity and decreases with increasing age. Girls are generally more hypermobile than boys and children from Asian backgrounds are generally more hypermobile than Caucasian children[5]

There is debate in the literature as to whether isolated joint hypermobility represents the end of the normal spectrum of joint range of movement or whether it represents a polygenic group at the mild end of the spectrum of Heritable Disorders of Connective Tissue[6,7]. It is generally accepted that the phenomenon runs in families and tends to be dominantly inherited. The diagnosis of generalised joint hypermobility, underlying genetic syndromes, and complications such as widespread musculoskeletal pain and chronic fatigue, are largely based on clinical criteria. The genetic causes of joint hypermobility include Ehlers – Danlos syndromes (EDS), some types of Osteogenesis Imperfecta (OI) including types I and IV, Marfan syndrome and related disorders, and rare HDCT such as pseudoxanthoma elasticum and cutis laxa syndromes. Hypermobility may also be a feature of a wide range of skeletal dysplasia syndromes eg pseudoachondroplasia and spondyloepiphyseal dysplasia congenita and developmental syndromes of childhood such as the Fragile-X syndrome.

Laboratory based genetic tests are available for some of the more severe types of EDS, OI and Marfan syndrome and are listed in tables 1, 2 and 3. Currently, confirmatory laboratory tests for the milder and commonly encountered HDCT's are not generally available or are prohibitively expensive. Clinical criteria are used to distinguish between Marfan syndrome (table 1), Ehlers-Danlos syndrome (table 2) and the milder forms of Osteogenesis Imperfecta (table 3). The Beighton score, which is the screening test for joint hypermobility, is incorporated into the diagnostic criteria for BJHS[8], EDS[9], Marfan[10] and OI[11]. EDS (hypermobile type) often becomes the "default" diagnosis if a hypermobile child does not meet the criteria for diagnosis of one of the other HDCT's and therefore this is probably a heterogeneous group. Patients with overlap conditions, for example OI/EDS, have been reported[12].

**Table 1:** Ghent Diagnostic Criteria for Marfan Syndrome – Ho[10].

System	Major criteria	Minor criteria
Skeletal system	Pectus carinatum Pectus excavatum requiring surgery Reduced upper to lower segment ratio or arm span to height ratio >1.05 Positive wrist and thumb signs Scoliosis of >20° or spondylolisthesis Reduced extension of the elbows (<170°) Medial displacement of the medial malleolus causing pes planus	Pectus excavatum of moderate severity Joint hypermobility High arched palate with dental crowding Facial appearance (dolichocephaly, malar hypoplasia, enophalmous, retrognathia, and down slanting palpebral fissures)

Ocular System	Ectopia lentis	Abnormally flat corneas Increased axial length of globe Hypoplastic iris or cillary muscles causing decreased miosis

Cardiovascular system	Dilation of the ascending aorta with or without aortic regurgitation and involving the sinuses of valsalva Dissection of the ascending aorta	Mitral valve prolapse with or without mitral valve regurgitation Dilatation of the main pulmonary artery

Pulmonary system	None	Spontaneous pneumothorax Apical blebs (shown on chest radiograph)

Skin and integument	Lumbosacral dura ectasia by CT or MRI	Stretch marks Recurrent or incisional herniae

Family history	Having a parent, child or sibling with either: -presence of a mutation in FBN1known to cause Marfans syndrome or -presence of a haplotype around FBN1, inherited by descent, known to be associated with Marfan syndrome in the family.	

For the proband the diagnosis requires the presence of major criteria in at least two organ systems and involvement of a third organ system. For a family member, diagnosis requires the presence of one major criterion in family history and one major criterion in an organ system and involvement of a second organ system.

**Table 2:** Diagnostic criteria for Ehlers-Danlos Syndromes – Beighton[9].

Type and Inheritance	Major features	Minor features	Laboratory
Classical AD	Skin hyperextensibility **Widened atrophic scars** Joint hypermobility	Smooth velvety skin Molluscoid pseudotumors Subcutaneous spheroids Complications of joint hypermobility (sprains, subluxations/dislocations, pes planus) Muscle hypotonia Delayed gross motor development Easy bruising Manifestations of tissue extensibility and fragility† Postoperative hernia Positive family history	Abnormalities in skin collagen under electron microscopy Abnormal collagen type V **30% due to mutation in tenascin**

Hypermobility AD	Skin involvement (hyperextensibility and/or smooth, velvety skin) Generalised joint hypermobility	Recurring joint dislocations Chronic joint/limb pain Positive family history	

Vascular AD	Thin, translucent skin **Arterial/intestinal/uterine fragility or rupture** Extensive bruising Characteristic facial appearance	Acrogeria Hypermobility of small joints Tendon and muscle rupture Talipes equinovarus Early onset varicose veins Arteriovenous, carotid-cavernous sinus fistula Pneumothorax/pneumohaemothorax Gingival recession Positive family history Sudden death in close relatives	Abnormal type 3 collagen COL3A1 mutation

Kyphoscoliotic AR	Generalised joint laxity Severe muscle hypotonia at birth **Scoliosis at birth, progressive** Scleral fragility and rupture of the ocular globe	Tissue fragility, including atrophic scars Easy bruising Arterial rupture Marfan-like habitus Microcornea Radiologically considerable osteopenia Family history	Urinalysis for lysylpyridinoline and hydroxylysylpyridinoline

Arthrochalasia AD	Severe generalised joint hypermobility with recurrent subluxations **Congenital hip dislocation**	Skin hyperextensibility Tissue fragility, including atrophic scars Easy bruising Muscle hypotonia Kyphoscoliosis Radiologically mild osteopenia	Skin biopsy and demonstration of abnormal collagen type 1

Dermatosparaxis AR	Severe skin fragility Sagging, redundant skin	Soft doughy skin texture Easy bruising **Premature rupture of fetal membranes** **Large hernias (inguinal and umbilical)**	Demonstration of abnormal collagen 1 chains in skin

†this included hiatus hernia, anal prolapse, cervical insufficiency for a diagnosis a patient must have one or more of the major criteria and presence of minor criteria is "suggestive" of a diagnosis. Items in bold are distinguishing features of that particular subtype of Ehlers-Danlos syndrome.

**Table 3:** Disorders predisposing to bone fragility which can be associated with joint hypermobility Munns and Sillence[11].

**Type**	**Inheritance**	**Discriminatory features**	**Other features**	**Laboratory**
Osteogenesis imperfecta type I	AD	**Blue sclera** Hypermobility, especially of small joints **Wormian bones **(70% of subjects) Generalised osteopenia on DEXA	Kyphoscoliosis Arcus cornea Hearing impairment Metatarsus varus Easy bruising Fractures from minimal trauma Opalescent dentine	**Reduction in synthesis of type I procollagen** **Abnormalities in COL1A1**

Osteogenesis imperfecta type I with opalescent dentine	AD	As above with **opalescent dentine**		

Osteogenesis imperfecta type IV	AD	**White sclera** Fractures from minimal trauma Short stature **Wormian bones **in 50–70%	Progressive long bone deformity Joint hypermobility	**COL1A1 or COL1A2 mutations which reduce collagen stability.**

Osteogenesis imperfecta type IV with opalescent dentine	AD	As above with **opalescent dentine**		

Geroderma osteodysplasticum	AR	Osteopenia **Platyspondyly** Learning disability Joint hypermobility Skin hyperelasticity	Wormian bones	

Items in bold are distinguishing features of that particular subtype of OI

It remains unclear why some hypermobile children become symptomatic while others remain symptom-free[13]. There is need for a reliable way of identifying children with joint hypermobility who are at high risk of developing musculoskeletal complications so that education and therapeutic interventions can be targeted to this group before they become symptomatic or sustain injuries[14,15]. It is also important to accurately identify children who are at risk of catastrophic cardiac or vascular complications later on, for example, children with EDS (vascular type).

(Benign) Joint Hypermobility Syndrome (BJHS) describes the combination of joint hypermobility with associated symptoms such as chronic joint pain, back pain, joint subluxation and dislocations, soft tissue injuries, Marfan syndrome-like habitus and skin features. It is diagnosed using the 1998 Brighton criteria[8] (table 4). The Brighton criteria contain both phenotypic features of HDCTs and symptoms which are thought to be complications of joint hypermobility. They exclude children with a diagnosed HDCT from the diagnosis of (benign) joint hypermobility syndrome, and this approach is confusing for clinicians. A standard way of describing a child who has articular and extra-articular complications of joint hypermobility is needed irrespective of the underlying genetic diagnosis or clinical phenotype.

**Table 4:** The 1998 Brighton criteria for a diagnosis of Benign Joint Hypermobility Syndrome[8].

*Major Criteria:*
**1. Beighton Score of ≥ 4/9**
**2. Arthralgia for > 3 months in > 4 joints**

*Minor Criteria:*

**1. Beighton score of 1–3**
**2. Arthralgia in 1–3 joints**
**3. History of joint dislocation**
**4. Soft tissue lesions >3**
**5. Marfan-like habitus**
**6. Skin striae, hyperextensibility or scarring**
**7. Eye signs, lid laxity**
**8. History of varicose veins, hernia, visceral prolapse**

For a diagnosis to be made either both of the major criteria must be present or 1 major and 2 minor or 4 minor.

## Methods

**Figure 1:**
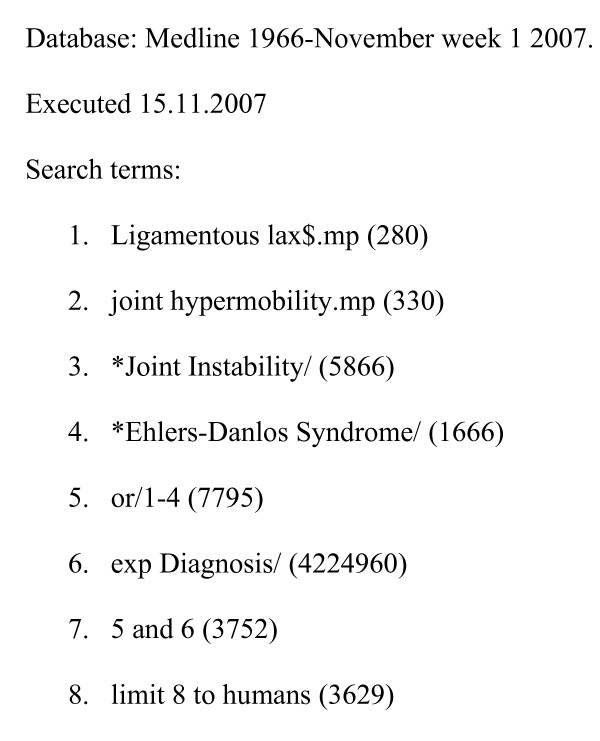
**Search strategy**.

Medline was searched using a strategy designed by a medical librarian to identify papers on the diagnosis of ligamentous laxity, hypermobility, hypermobility syndromes and related HDCT's (Figure 1). Duplicates were excluded and titles were hand searched. Papers which focused on joint stability following joint replacement surgery were excluded, leaving a total of 3330 papers. The titles of papers that focused on a single joint were checked to determine which joints were most frequently the focus of published works but not further analysed.

The conclusions of this literature analysis were compared with the experience of The Connective Tissue Dysplasia (CTD) Clinic, a multidisciplinary clinic established in 1987 at the Children's Hospital at Westmead, Sydney, Australia to coordinate the care of patient groups with HDCT.

### Search results

In total, 3629 papers were identified, of which 63 (which were duplicates) and 236 (which related to instability following joint replacement surgery) were excluded, leaving 3330 papers. Of these, 1534 papers focused on laxity of a specific joint: the majority related to the shoulder, ankle, spine, knee and TMJ as illustrated in table 5. Most of these papers describe joint laxity resulting from soft tissue injury rather than inherited joint laxity.

**Table 5:** Frequency of joints which are the main focus of papers on Ligamentous laxity

**Joint**	**Number of papers**	**Frequency %**
Temporomandibular joint	30	1.1
Spine	302	12
Shoulder	861	34.3
Elbow	225	9
Wrist	229	9
Hip	229	9
Knee	287	11.4
Patellofemoral joint	19	0.7
Ankle	325	13
**Total Single Joint**	**1534**	**99.5%**
**Ehlers-Danlos Syndrome**	**1666**	
**Joint Hypermobility**	**330**	

*Total*	***3330***	

The totals do not add up to 3330 as 200 papers discussed both instability of a single joint and either Ehlers-Danlos syndrome or Joint hypermobility syndrome.

There were 1666 papers relating to the diagnosis of Ehlers-Danlos syndromes and a further 330 to joint hypermobility syndrome(s). The two articles which focus on the validity of diagnostic criteria are both written by Remvig[5,16] and represent comprehensive reviews of the current literature on the diagnostic tests for identifying generalised joint hypermobility and the criteria for diagnosing Benign Joint Hypermobility Syndrome (BJHS).

#### Definitions of hypermobility, hyperlaxity, hyperextensibility

The words "hypermobility", "hyperlaxity" and "hyperextensibility" are used interchangeably by some authors[17] and have not been clearly defined. The word "hypermobility" is most commonly used to describe excessive movement in the normal plane of movement, most frequently hyperextension, and "laxity" is used to describe excessive movement in an abnormal plane of movement e.g. inferior subluxation of the shoulder giving an inferior sulcus sign. However, joints can be hypermobile without being lax and we believe that whereas laxity is a more important predictor of instability of any particular joint, generalised hypermobility, weakness and poor proprioception are better predictors of generalised symptoms such as widespread arthralgia and fatigue.

### The relationship between Benign Joint Hypermobility Syndrome and Heritable Disorders of Connective Tissue

The Brighton criteria (table 4) for the diagnosis of BJHS include phenotypic features eg "Marfanoid Habitus" as well as symptoms and Remvig argues that use of "Marfan-like habitus and eye signs in the diagnostic criteria is questionable". The original Brighton criteria[8] stated that a diagnosis of BJHS could only be made in the absence of a diagnosed HDCT. However we find in clinical practice that patients with HDCTs frequently have widespread arthralgia and/or disabling fatigue and/or joint instability episodes and need rehabilitation for these symptoms as well as genetic and medical management for their underlying condition.

**Figure 2:**
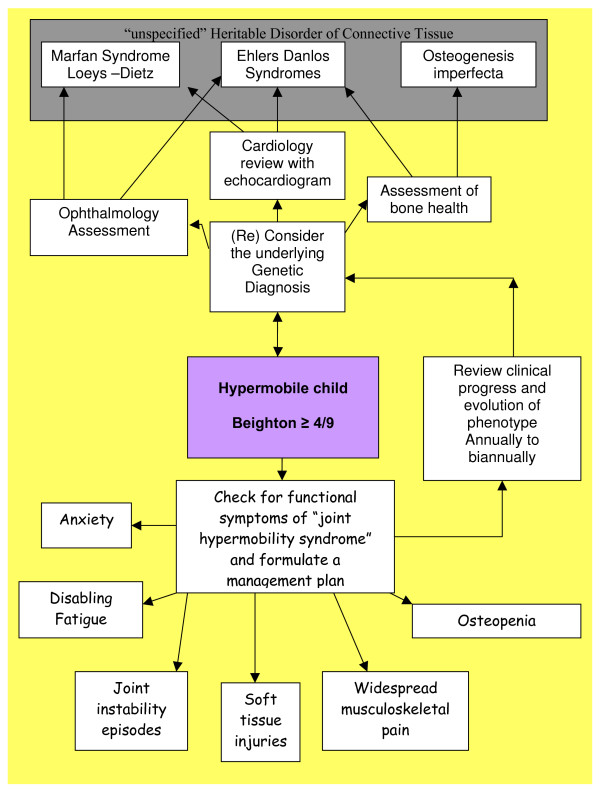
**The connective tissue dysplasia clinic diagnostic approach to a child with hypermobile joints**.

Our clinical approach to children presenting with joint hypermobility is illustrated in figure 2. We work as a team with clinical geneticists establishing the diagnosis of the underlying HDCT where possible and the interdisciplinary rehabilitation team identifying and managing related functional symptoms.

### Distinguishing between Ehlers-Danlos syndrome (hypermobile type) and "normal spectrum hypermobility"

There is a spectrum of generalised joint hypermobility in children and the phenomenon is almost certainly polygenic in origin with environmental influences, particularly participation in sport and flexibility training. Younger children are more flexible but this resolves with increasing age in normal children. It is a clinical challenge to distinguish young children with significant hypermobility who are unlikely to improve from those who are in the normal spectrum of hypermobility and will improve with time. Clinical follow up over several years is currently the only way of answering this question for an individual child.

The current criteria for a diagnosis of Ehlers-Danlos syndrome (hypermobile type) are illustrated in table 2 but are non-specific. For a diagnosis to be made an individual needs to have one of the major criteria of a Beighton score of ≥ 4/9 **or **"Skin involvement (hyperextensibility and/or smooth, velvety skin)". Beighton et al[9] describe testing of skin hyperextensibility at a neutral site e.g. the volar aspect of the forearm where "the skin is pulled up until resistance is felt" but they do not describe a reproducible measurement which can be taken during this test, nor what might be normal and abnormal. Minor criteria which are "of lesser diagnostic specificity" include recurrent joint dislocation, chronic joint/limb pain and a positive family history. The presence of one or more minor criteria is "suggestive" of the diagnosis. The criteria are much clearer than in previous classifications for the more severe subtypes of EDS, but are not specific enough to be very helpful in distinguishing between EDS (hypermobile type) and "normal spectrum" hypermobility. We normally use the diagnosis of Ehlers-Danlos syndrome (hypermobile type) in patients with one major criteria and any one of the minor criteria and tend to diagnose individuals whom we perceive to have very soft skin as EDS (hypermobile type), although we do not have an objective measure of skin quality.

We refer all our hypermobile patients with a Beighton score of ≥ 4/9 for a cardiac and echocardiography assessment to look for mitral valve and aortic root abnormalities. If minor cardiac signs are present we use the EDS (hypermobile type) diagnosis[18] and consider the diagnosis of Marfan syndrome.

Identifying the subgroup who have more muscle involvement e.g. those with tenascin-X deficiency[19], is particularly relevant to rehabilitation management because there is a subgroup of children who respond poorly to muscle strengthening interventions and need more intensive rehabilitation over a longer period to achieve their functional goals. We do not currently test for tenascin-X deficiency in clinical practice.

### Distinguishing between Ehlers-Danlos syndrome (hypermobile type) and Osteogenesis Imperfecta types I and IV and the evaluation of bone health in hypermobile children

Children with mild subtypes of Osteogenesis Imperfecta (OI) are usually of average stature and usually have a history of minimal trauma fractures. Patients may also have mid thoracic back pain from vertebral crush fractures. We have, however, identified family members with OI type I who have never sustained a fracture (see Appendix 1). Dark blue sclerae are a distinguishing feature of OI type I as is opalescent dentine (dentinogenesis imperfecta) and Wormian bones are highly suggestive but not pathognomic of OI. In one clinical study of 65 patients with OI type I, 64.6% had hypermobility in the upper limbs and 36.9% in the lower limbs evidenced by genu recurvatum[20]. Seventy percent of patients with OI type I in this study had recurrent sprains and dislocations did occur but were uncommon. Easy bruising was reported in 78% of subjects and was frequently present at interview. Osteogenesis imperfecta is a disorder of high bone turnover so markers of bone turnover such as urinary deoxypyridinoline/creatinine ratio are frequently elevated.

Children with mild OI have slender bones, increasing their risk of fracture. Due to decreased bone size, bone mineral content (BMC) is reduced and there is a reduction in the BMC for lean tissue mass ratio, reflecting the primary nature of the bone pathology. Dual energy x-ray absorbtiometry (DXA) can be used to measure these parameters but accurate interpretation depends on the availability of age matched standards. Another useful and accessible tool for assessing bone phenotype is the comparison of the cortical diameter of the 2^nd ^metacarpal bone to normal values[21].

Patients with generalised joint hypermobility have been shown to be at risk of osteopenia in several studies[17,22-24] so it is worthwhile evaluating bone health in all hypermobile children. Gulbar[23] demonstrated a correlation between increasing Beighton score and decreasing bone density at the hip, measured by DEXA, in a small case-control study of hypermobile adult women after controlling for confounders such as smoking and physical activity. The osteopenia is most likely to be a result of muscle weakness and lack of constraint to joint movement, which decrease the forces transmitted to the bone. It is possible that some undiagnosed OI patients were included in some of the above studies, affecting the results. We recommend investigating all patients with a significant fracture history, blue sclera and/or Dentinogenesis Imperfecta for Osteogenesis Imperfecta and also evaluate bone health in children with a Beighton score of ≥ 4/9 using the investigations listed in Appendix 2.

### Distinguishing between Ehlers-Danlos (Hypermobile type) and Marfan syndrome and related disorders

Marfan syndrome is well described and the available algorithm for diagnosis (table 1) is based on the clinical features. The reliability and validity of the Ghent criteria has not been established. Marfan syndrome is caused by mutations in the FBN1 gene which codes fibrillin, a structural protein present in microfibrils and a key component of connective tissue. In a recent cohort study of patients with a laboratory confirmed FBN1 mutation, 600/956 (63%) had "ligamentous laxity", which was the third commonest major skeletal feature after arachnodactyly and high arched palate[25]. Clinically, we have found that some hypermobile patients with a "Marfan-like" habitus do not meet the criteria for a formal diagnosis of Marfan syndrome. It is wise to be cognisant of the fact that the phenotype will evolve with time and that finding a FBN1 mutation in a child with major skeletal features would support a diagnosis. For example, a child with pes planus and mitral valve prolapse would meet the criteria for a diagnosis of Marfan syndrome if a FBN1 mutation was found on testing. Children who have a mutation but do not meet the Ghent criteria are described as having a type 1 fibrillinopathy. There is an increased incidence of Marfan-like habitus in some hypermobile populations[7] but the influence of this feature on the risk of complications of hypermobility is unclear.

Marfan syndrome can be complicated by chronic widespread musculoskeletal pain and fatigue and patients with Marfan syndrome may also meet the diagnostic criteria for (benign) Joint Hypermobility Syndrome and/or chronic fatigue syndrome. The latter group of patients may have very disabling symptoms and a similar rehabilitation approach to that used for other hypermobile children can be used, tempered in its intensity by the patient's cardiovascular involvement. Using a diagnosis of Joint Hypermobility Syndrome in symptomatic patients with Marfan syndrome aids in defining and understanding a particular patient's needs.

### Vascular involvement – Ehlers Danlos Syndrome (Vascular type) or Loeys-Dietz Syndrome?

There are subgroups of children who present with hypermobility and who are at significant risk of mortality as a result of vascular fragility in early adulthood. These children have hypermobility, particularly of small joints, thin translucent skin, lack of subcutaneous fat and bruise easily. They are prone to complications including aortic dissection, stroke from ruptured cerebral vessels and uterine and bowel rupture. There may be a family history of sudden death. EDS (vascular type) is due to a defect in COL3A1 therefore a diagnosis can be made if mutations are found in the type III collagen gene.

As with the other HDCTs phenotypic overlap exists between EDS (vascular type) and another disorder, Loeys-Dietz syndrome. This is a recently described autosomal dominant syndrome characterised by arterial tortuosity, hypertelorism and bifid uvula or cleft palate. It is caused by mutations in the transforming growth factor beta receptor genes (TGFBR1 and 2). The disorder is characterised by arterial aneurysms and mean age of death in a recent cohort was 26 years[26]. The authors comment that one way of distinguishing between the two diagnoses is the lower rate of intraoperative mortality in Loeys-Dietz syndrome – not an ideal diagnostic test. As this syndrome has been described so recently the cases identified have been those presenting with complications. Mildly affected relatives who have been identified as having the genetic mutation after families have been screened may present with isolated hypermobility, especially in childhood[26].

## Conclusion

We describe our approach to the diagnosis of a child who presents with hypermobility and related symptoms. It is important to consider the child's underlying genetic diagnosis as well as to accurately describe their functional symptoms. This results in some children having two diagnoses – one of their HDCT and another describing functional complications. As the field of genetics moves forward we will be able to more accurately diagnose the genetic disorder underlying an individual child's hypermobility and the relevance of subtle phenotypic features in the mild HDCT will become clearer.

We believe that the "benign" descriptor in "benign joint hypermobility syndrome" is misleading and unhelpful and prefer "Joint Hypermobility Syndrome" to describe the combination of generalised joint hypermobility and functional symptoms. Consideration needs to be given to limiting diagnostic criteria to symptoms only rather than including a mixture of phenotypic features which are probably more relevant to the underlying genetic diagnosis.

Most authors now agree that children diagnosed as having Ehlers-Danlos syndrome (hypermobile type) or (benign) joint hypermobility syndrome represent the mild end of the spectrum of heritable disorders of connective tissue. Current research using this group is challenging because there is significant variability between individuals in this ill-defined population and some have features which overlap diagnostic groups. There is a need for descriptive studies of large cohorts of individuals with joint hypermobility to investigate the relationship between baseline clinical characteristics and adverse outcomes. In our experience this condition can have a significant negative impact on an affected child's ability to function and participate in society and is much more disabling than generally recognised. Accurate diagnosis of Joint Hypermobility Syndrome facilitates early referral to an interdisciplinary team with appropriate clinical expertise in its management and avoids the use of ineffective measures, in particular the over prescription of analgesia.

It is important to recognise and manage functional symptoms in all children with Heritable Disorders of Connective Tissue.

## Competing interests

The authors declare that they have no competing interests.

## Authors' contributions

LT designed the study, carried out the literature review and drafted the manuscript. EE participated in the design of the study and edited the manuscript. CM provided significant input to the parts of the manuscript discussing Osteogenesis imperfecta and edited the manuscript. VP contributed to the design of the study and analysis of the literature and helped draft the manuscript. DOS helped draft the manuscript and provided senior clinical and genetic opinion. All authors read and approved the final manuscript

## Appendix 1

### Case study – AM

AM is an 11 yr old girl who presented with joint hypermobility (Beighton score 7/9) and instability episodes. She was initially diagnosed as having Ehlers-Danlos Syndrome (hypermobile type). She has dark blue sclera and a family *but not a personal *history of low trauma fractures. Osteopenia compared to age matched controls was present on DEXA so her diagnosis was revised to Osteogenesis Imperfecta type 1.

AM's current clinical problems include:

• recurrent dislocation of the right 4^th ^metacarpophalyngeal joint

• painful toe subluxations

• longstanding anterior knee pain worse after activity

• handwriting difficulties

Although OI is her genetic diagnosis, her symptoms and functional problems relate to her Joint hypermobility and management needs to be focused on these areas. She would meet the Brighton criteria for a diagnosis of benign joint hypermobility syndrome but is excluded from this group as she has mild OI. Her management ideally should include multidisciplinary musculoskeletal rehabilitation.

## Appendix 2

### Assessment of bone health in hypermobile children

Blood tests:

• Urea and Electrolytes

• Calcium magnesium and phosphate

• Alkaline phosphatase

• Parathyroid Hormone

• Full blood count and ESR

• Osteocalcin

• Vitamin D (25 hydroxyvitamin D)

• Thyroid Stimulating Hormone

• Oestradiol

• Testosterone

• Antitissue Transglutaminase antibodies

• Immunoglobulin A

Urine Tests

• Random urine creatinine

• Random urine CA:CR ratio

• Deoxypyridinoline and Deoxypyridinoline:CR ratio

• Urine metabolic screen

DEXA scan for Bone Mineral Density

### Additional investigations to look for Osteogenesis Imperfecta

Evaluation of cortical thickness from anteroposterior hand and wrist ("bone age") radiographs

1. measure total bone diameter of second metacarpal on the right hand at the midpoint of the bone

2. measure trabecular bone diameter

3. calculate ratio

4. compare to normal values for age and sex as published in Spencer et al

Skull X-ray to look for Wormian Bones (more than 10 which are of greater than 4 × 6 mm in size).
